# Barriers to and Facilitators of Childhood Immunization in Central Ethiopia Region: A Qualitative Study

**DOI:** 10.1002/puh2.70456

**Published:** 2026-11-07

**Authors:** Zeleke Dutamo Agde, Temesgen Abdi, Tesfaye Lejisso, Samuel Darge, Ashenafi Petros, Temesgen Kelaye, Legese Petros, Mamush Hussein, Belayneh Hamdela Jena, Amanuel Nuramo

**Affiliations:** ^1^ Department of Public Health College of Medicine and Health Sciences Wachemo University Hosaina Ethiopia; ^2^ Central Ethiopia Regional State Health Bureau Worabe Ethiopia; ^3^ Central Ethiopia Regional State Public Health Institute Worabe Ethiopia; ^4^ Department of Midwifery College of Medicine and Health Sciences Wachemo University Hosaina Ethiopia

**Keywords:** barriers to immunization, central Ethiopia, childhood immunization, facilitators to immunization, under‐immunized, zero‐dose children

## Abstract

**Background:**

Childhood vaccination remains one of the most successful and cost‐effective public health interventions, preventing millions of illnesses and deaths each year. Ethiopia is one of the countries with a high number of zero‐dose children and under‐immunization. However, there is limited evidence about the barriers to and facilitators of immunization. Therefore, this study aimed to explore the barriers and facilitators of immunization in central Ethiopian region.

**Methods:**

An exploratory qualitative study was employed to collect data from October 15, 2025, to December 14, 2025. A total of 109 participants were involved in the qualitative study. We employed a purposive sampling method to select study participants. Data were collected through focus group discussions (FGDs), in‐depth interviews (IDIs), and key informant interviews (KIIs) guided by a semi‐structured topic guide. Data were analyzed using thematic analysis approach.

**Results:**

Three themes emerged from the data. The themes included awareness of child immunization and facilitators and barriers of child immunization. Positive attitude and beliefs toward immunization, family and social support, and mothers’/caregivers’ motivation and responsibility were found to be the facilitators of immunization, whereas fear and misconceptions related to vaccines, lack of awareness and misinformation, health‐system and access barriers, and competing priorities and workload were mentioned as the barriers to immunization.

**Conclusions:**

This study highlights that childhood immunization uptake in central Ethiopia is shaped by a complex interaction of individual, social, and health‐system factors. Positive maternal attitudes and beliefs, strong family and community support, and caregivers’ sense of responsibility emerged as key facilitators of immunization. In contrast, fear and misconceptions about vaccines, inadequate awareness and misinformation, health‐system and access constraints, and competing household responsibilities were major barriers. Interventions that strengthen community awareness, counter misinformation, enhance family and social support, and improve the accessibility and responsiveness of immunization services are helpful to improve the immunization uptake.

## Graphical abstract

Childhood immunization uptake in central Ethiopia is influenced by individual, social, and health‐system factors. Addressing misinformation, strengthening family and community support, and improving access to immunization services are essential to increase vaccination uptake.

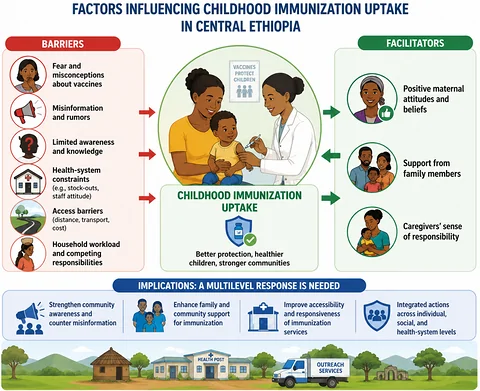

## 1 Introduction

Childhood immunization is among the most impactful and cost‐effective global health interventions, saving millions of lives each year [1]. “Zero‐dose (ZD) children” are those children who did not receive the first dose of diphtheria‐tetanus‐pertussis (DPT1) containing vaccine, and under‐vaccinated children are those who did not receive the third dose of (DTP3) containing vaccine by the end of their first year of life [2, 3].

According to a WHO report, about 14.3 million children have zero doses. Of these, almost all burdens are concentrated in low‐ and middle‐income countries (LMICs), especially sub‐Saharan Africa and Southeast Asian regions. Although substantial progress has been made in childhood immunization over the past two decades, Ethiopia remains one of the 10 countries that account for more than half (55%) of all ZD children worldwide [4].

ZD children are at increased risk of infections with vaccine‐preventable diseases (measles, polio, and pneumonia) during the first 5 years of life [5, 6]. Strengthening children's access to basic interventions such as vaccination is a key strategy in improving child health [7]. Childhood immunization reduces morbidity and mortality related to vaccine‐preventable diseases [8]. The Expanded Program of Immunization (EPI) has averted an estimated death of 154 million children globally since the inception (1974) [9].

Findings have shown that the prevalence of ZD children in Ethiopia is one of the highest in the world, ranging from 10% to 46.5% with significant spatial variation across the country [10, 11, 12]. A recent study of underserved and special setting populations in Ethiopia found a 33.7% prevalence of ZD children [5].

Literature has shown that destruction of health‐system infrastructure due to conflict or war, internal displacement, and vaccine supply disruption has impacted the vaccination program [13, 14]. Moreover, studies conducted in Ethiopia have shown that rural residence, being uneducated, home delivery, poor wealth index, no antenatal care (ANC) visits, and low media exposure were found to be positively associated with ZD children and under‐immunization [5, 11, 12, 15]. In Ethiopia, childhood vaccine hesitancy has been associated with traditional beliefs and gender norms. A study conducted in four regions of Ethiopia (Amhara, Oromia, Afar, and Tigray) has found that about 51% of the caregivers showed some degree of vaccine hesitancy [16].

This study's findings help to achieve Sustainable Development Goal 3, aiming to reach the Immunization Agenda 2030 (IA 2030) target to halve the number of ZD children by 2030 [17]. Several studies conducted in Ethiopia addressed the immunization coverage and associated factors, mainly from demand‐side perspectives [5, 11, 12, 15]. However, limited evidence exists on context‐specific barriers and facilitators of childhood immunization from multiple perspectives in the Central Ethiopia region. Therefore, this study aimed to explore the barriers and facilitators of childhood immunization from the perspectives of mothers, caregivers, community leaders, and health‐system stakeholders in central Ethiopia.

## 2 Methods and Materials

The study was conducted in the central Ethiopia region. The region is located in the southwestern part of Ethiopia, between 70°04′ and 80°03′ north latitude and 37°20′ and 38°60′ east longitude. The central Ethiopia region shares borders with the Oromia region in the north, west, and east; the South Ethiopia region in the south direction; and the Southwest Ethiopia People's Region in the southwest direction. It had a population of about 7.2 million in 2025. It also shows that 47.9% and 48.3% of the total population are less than 15 years old and 15–65 years old, respectively. The region consists of 7 zones and 3 special woredas with 55 woredas and 27 urban administrations. The region has 1294 kebeles, of which 185 are urban and 1109 are rural. At the end of the 2024 fiscal year, 1067 health posts, 229 health centers, and 30 hospitals (2 comprehensive, 4 general, 24 primary levels, 4 non‐governmental, and 1 private) were providing services to the public. The total number of health sector workers in the region is about 13,995. The study was conducted from October 15 to December 14, 2025.

### 2.1 Epistemological Perspectives

This study was guided by an interpretivist paradigm, which assumes that reality is socially constructed and that individuals understand and interpret phenomena based on their experiences and social contexts [18]. This approach was considered appropriate because the study sought to explore the barriers and facilitators of childhood immunization from different perspectives such as mothers/caregivers, male partners, and immunization stakeholders.

### 2.2 Study Design/Approach

The study employed an exploratory qualitative study design to explore the facilitators and barriers of child immunization in the Central Ethiopia region. This study design was chosen to uncover deep insight from different perspectives and identify emerging patterns [19].

### 2.3 Study Participants

Study participants from both supply and demand sides were selected and considered. Study participants were selected by using purposive sampling techniques. The supply side involved participants from regions, zones, and woredas who were decision‐makers, directors, coordinators, or facilitators of the vaccination program. From the demand side: mothers/caregivers in in‐depth interviews (IDIs). Mothers/Caregivers ZD children (children who did not receive the first dose of diphtheria‐tetanus‐pertussis‐containing vaccine) aged 12–59 months that were identified by pre‐survey were included in the study. Mothers who were severely ill at the time of data collection were used as exclusion criteria.

Religious leaders, community leaders, and the Women Development Army (WDA) were involved in focus group discussions (FGDs). Influential members of the community who served or lived in the community at least 6 months were included in the study, whereas those unable to communicate effectively due to severe illness were excluded.

From the supply side, decision‐makers, directorates, coordinators, or facilitators who were working on activities related to the vaccination program at the regional, zonal, and woreda levels were involved in key informant interviews (KIIs).

### 2.4 Data Collection

A semi‐structured topic guide was used to collect data. It was developed by reviewing similar studies in the literature. The tool was translated from English to Amharic (official language) by bilingual translators. Then, it was translated back to ensure the conceptual equivalence. The data collectors and supervisors received 3 days of intensive training on the study objectives, qualitative research principles, ethical considerations, facilitation of FGDs, IDI techniques, effective probing, note‐taking, audio recording procedures, and strategies. The translated version was pre‐tested with five mothers with ZD children aged 12–59 months. Then, amendments were made accordingly. Data were collected by public health professionals with master's degrees who had prior experience in qualitative data collection. Data were collected by taking notes and using an audio recorder after getting permission from the study participants. Mothers/caregivers with ZD children were considered for IDIs to gain a deeper understanding of barriers and facilitators of immunization. The moderator and note–taker, who had previous experience, conducted all FGDs. All FGDs, IDIs, and KIIs were conducted in a safe and private environment. About 45–60 min were spent for all IDIs, making an average of 50 min. For KIIs, 40–45 min were spent, making an average of 42 min. Moreover, 55–65 min were utilized for FGD, making an average of 55 min. Participants of the FGD were arranged in a semi‐circle position to enhance open communication, ensure clear visibility among participants, and facilitate active engagement and interaction during the discussions [20]. The ground rules and regulations were made prior to the interviews and discussions. All FGDs were conducted at health posts, whereas KIIs and IDIs were conducted at their workplace (office) and home, respectively.

A semi‐structured topic guide was developed by reviewing similar studies [21, 22, 23]. It included the awareness of childhood immunization, barriers, and facilitators to childhood immunization. During the discussions and interviews, each primary question was supplemented with follow‐up questions and probing techniques to obtain a deeper understanding of barriers and facilitators to childhood immunization [20].

### 2.5 Data Processing and Analysis

Prior to data analysis, the transcriptions were carried out by taking into consideration audio recordings and field notes. Then, it was imported to ATLAS.ti 7.1 software for analysis. Transcripts were read line by line to gain a deeper understanding of the content, identify meaningful units, and ensure familiarity with participants’ experiences and viewpoints. Braun and Clarke's thematic analysis framework, employing an inductive approach, was used for data analysis [24]. The data were initially coded independently by two coders. Subsequently, consensus codes were developed through agreement between the two coders. The two coders discuss discrepancies and resolve differences through consensus. These codes were then grouped into categories and overarching themes. All the themes and categories were critically reviewed to capture the content and meaning of the data. The findings were synthesized and presented, supported by relevant illustrative direct quotations.

### 2.6 Trustworthiness

The following four key issues were addressed to assure the trustworthiness of the study. Credibility of the study was assured through peer debriefing, member checking, and data from multiple sources (mothers with ZD children, health decision‐makers related to vaccination, religious workers, and community leaders were triangulated). Likewise, thick description of methods, selection of study participants, and data collection and analysis were carried out. To enhance the transferability of this study, a comprehensive and detailed explanation of the research process, including methodology, participants’ selection and recruitment and sampling techniques, result interpretation, and the contribution of researchers, will be clearly explained in a thick description [25]. In order to ensure dependability, research context, selection, and recruitment of the participants; data collection; and analysis procedures were also comprehensively described. Confirmability was ensured through peer debriefing, member checking, and an audit trail [25].

## 3 Results

### 3.1 Participant Characteristics

A total of 10 FGDs with 91 participants, 10 KIIs, and 8 IDIs were involved in this study. Overall, a total of 109 participants were involved in the study. Three invited FGD participants and one KII participant declined to participate. Total participants in FGDs, IDIs, and KIIs were determined due to data saturation; no new information emerged (Table 1).

**TABLE 1:** Characteristics of study participants in focus group discussions, in‐depth interviews, and key‐informant interviews, Central Ethiopia Region, December, 2025.

Participants	Data collection method	Age range	Average age	Number of participants	Number of FGD/KII/IDI
Mothers	FGD	18–42	29	91	10
Health decision‐makers	KIIs	25–52	38	10	10
Mothers/Caregivers of zero‐dose children	IDI	19–40	28	8	8
Total				109	28

Abbreviations: FGD, focus group discussion; IDI, in‐depth interview; KII, key informant interview.

The thematic analysis approach was employed for data analysis. Therefore, 46 codes, 11 categories, and 3 themes emerged from the data. The themes included awareness of child immunization and facilitators and barriers of child immunization (Table 2).

**TABLE 2:** Themes and categories of barriers to and facilitators of childhood immunization in Central Ethiopia, December, 2025.

Categories	Themes
Awareness of purpose and benefits of immunization	Awareness on child immunization
Awareness on immunization schedule	
Awareness of vaccine safety and side effects	
Awareness of source of immunization information	
Positive attitudes and beliefs toward immunization	Facilitators of child immunization
Family and social support	
Health‐system support	
Mothers/Caregivers motivation and responsibility	
Fear and misconceptions related to vaccines	Barriers of immunization
Lack of awareness and misinformation	
Health‐system and access barriers	
Competing priorities and workload	

### 3.2 Theme 1: Awareness of Child Immunization

#### 3.2.1 Awareness of Purpose and Benefits of Immunization

Study participants in FGDs and IDIs mentioned that vaccines are important because they prevent diseases and serious illness.

Whenever a new child is born into our family, I immediately think about immunization because I know it protects children from many diseases (FGD, 26‐year‐old mother). Additionally, vaccination is important for a child's health; it protects those [children] from disease (IDI, 32‐year‐old mother).

However, the contrasting understanding was observed among IDI participants. Some participants in IDI revealed that although they know what the immunization is, they did not know the benefit of the immunization; rather, they perceived that the immunization brings sickness to the child. The study participants misperceived that vaccination might result in some illness in children when they got vaccinated.

I do not trust the vaccines given to children. I have observed that children who receive vaccines often become febrile, lethargic, and restless. Because of this, I do not want to bring such problems to my child by allowing immunization (FGD participant, 36‐year‐old community leader).

#### 3.2.2 Awareness on Immunization Schedule

Mixed results were explored in awareness of immunization schedules. Participants mentioned that they were aware of the immunization schedule, whereas some of the FGD and IDI participants stated a lack of knowledge on the timing of the immunization schedule.

I did not know when start the immunization and when to finish it (IDI, 25 years old mother).

#### 3.2.3 Awareness of Vaccine Safety and Side Effects

The study participants were aware of side effects of the vaccination, such as mild fever, pain/swelling, and restlessness, and they noted that these side effects typically go away after 2 or 3 days. On the contrary, some participants mentioned that they did not bring their newborn to a health facility due to fear of the side effects.

…After vaccination there may be fever and pain, but they go away after two or three days. That is why some mothers do not get their children vaccinated. That is wrong; vaccines are safe, not fatal (FGD, 24‐year‐old mother).

#### 3.2.4 Awareness of the Source of Immunization Information

The commonly mentioned sources of information about immunization/vaccination were health extension workers (HEWs) and health care facilities, such as health posts, health centers, and hospitals.

### 3.3 Theme 2: Facilitators of Child Immunization

#### 3.3.1 Positive Attitudes and Beliefs Toward Immunization

Study participants mentioned that the trust in the vaccines and its importance motivated their child to get vaccinated in addition to government support and efforts to get all eligible children to get fully vaccinated.

In addition to the government's stamina, the community's positive attitude plays a key role in shaping the mothers'/caregivers' attitude towards immunization (KII, 36‐year‐old MCH coordinator). Added, vaccines are one of the best things for children; they grow strong and healthy when they get vaccinated (IDI, 30‐year‐old mother).

#### 3.3.2 Family and Social Support

The study revealed that the male partners’ support and encouragement played a great role in getting child immunization. The participants reported that encouragements, reminders, and financial support helped them to get child vaccinations.

My husband reminds and motivates me to go to the health center and gives money for transport to vaccinate the child. Without her husband's support, it was not possible to handle everything concerning vaccination. We [mothers] are always busy with household chores (FGD, 32‐year‐old mother).

#### 3.3.3 Health‐System Support

The study participants mentioned that the reminders about the vaccination and the vaccination schedules from HEWs also motivated them to get the child vaccinated. The participants in FGDs mentioned that health education given during community gatherings and health centers facilitated them to vaccinate their child.

Health extension workers usually inform us about the benefits of vaccination, the correct schedule, and the possible consequences if a child is not vaccinated. They explain how vaccines protect children from serious diseases and remind us when to return for the next dose, which helps us not to miss appointments (FGD, 29‐year‐old mother).

#### 3.3.4 Mothers’/Caregivers’ Motivation and Responsibility

In this study, participants in FGDs have revealed that the vaccination of children is the responsibility of the mothers or caregivers. Also added, keeping the health of one's child was considered the sole responsibility of the mother.

### 3.4 Theme 3: Barriers to Child Immunization

#### 3.4.1 Fear and Misconceptions Related to Vaccines

Fear of vaccination side effects emerged as a key barrier to child immunization. Participants described concerns related to post‐vaccination fever, pain, and persistent crying among children.

Sometimes, children develop fever and pain after vaccination, and there are occasions when they cry continuously, which makes mothers afraid to return for the next dose (FGD participant, 36‐year‐old mother).

#### 3.4.2 Lack of Awareness and Misinformation

Lack of awareness and misinformation were identified as one of the key barriers to childhood immunization in the qualitative study. Some participants perceived vaccines as harmful, associating them with adverse effects such as fever, loss of appetite, and restlessness, which contributed to vaccine hesitancy. This might also interact with fear of side effects, low educational status, and inadequate exposure to information related to child vaccination, leading to reduced confidence and no or delayed or under‐immunization.

I am afraid to vaccinate my child because I have seen children become sick after receiving vaccines. They develop fever, stop eating, and become very restless. Because of this, I feel the vaccine may do more harm than good (IDI, 30 years old mother).

#### 3.4.3 Health‐System and Access Barriers

Long distances to nearby health facilities such as health posts, health centers, and hospitals and difficulty to reach were reported as barriers to child immunization. The long travelling time increased transportation and opportunity costs, which discouraged the mothers or caregivers from attending immunization schedules at health institutions.

The main reason why my child has not received any vaccination is the distance to the health facility and lack of awareness. She added, “There have been times I wanted to vaccinate my child, but the distance to the health post was too far” (IDI, 26‐year‐old mother with child zero‐dose).

#### 3.4.4 Competing Priorities and Workload

Study participants mentioned that heavy household chores and income‐generating activities hindered taking the children to get vaccinated. Mothers and caregivers, particularly in rural settings, often experience a heavy workload that leaves them overwhelmed with competing domestic, agricultural, and caregiving responsibilities.

## 4 Discussion

In this study, participants mentioned that there is a lack of awareness of the benefits of vaccination of a child. Likewise, fear of side effects let them not get vaccinated for their children. Existing literature has shown that fear of side effects and poor maternal knowledge of immunization positively affected child immunization [26]. False beliefs that vaccination may result in illness and physical impairments, such as paralysis, were found to be one of the misconceptions expressed by the study participants. Vaccine hesitancy may be due to negative beliefs based on myths, misinformation, and mistrust of health care providers. Vaccine hesitancy was considered one of the 10 threats to health by the WHO in 2019 [27, 28]. Similarly, existing literature in Ethiopia has evidenced that vaccine hesitancy is positively associated with untimely or no vaccination [16, 29]. This emphasizes an intervention that focuses on raising awareness of vaccine importance and safety and benefits and addressing the challenges of vaccine hesitancy, which positively influences immunization.

Several facilitators to immunization were explored in this study, such as positive attitude, support from the family, home visits by HEWs, and caregivers’ motivations. Existing evidence has shown that positive attitudes and beliefs toward immunization positively influence the immunization [30, 31]. Moreover, male partners often act as key decision‐makers for children receiving vaccinations, providing money for transportation and enabling the women to access the health care facilities. A study conducted in the Bibugn district of Ethiopia has shown that less than 50% of the male partners were involved in the immunization of the child [32]. Tailored, community‐based awareness programs delivered through trusted platforms such as health extension HEWs, community leaders, and women's groups can help reinforce favorable attitudes and sustain demand for immunization services. The family involvement, specifically of male partners, indicates a desire to shift from mother‐only approaches to more family‐centered or couple‐based interventions [33].

In this study, participants mentioned that remoteness in terms of the distance to the nearest health care facilities was a major obstacle to vaccination uptake. This corroborates other observational study findings [34, 35]. Long distance to health facilities likely increases transportation costs, travel time, and opportunity costs, particularly for mothers/caregivers whose daily livelihoods depend on household chores or other labor‐intensive activities. This may discourage attending the immunization schedules. Ethiopia has made tremendous effort in expanding primary health care and the number of rural and urban HEWs over the last 15 years to mitigate such challenges [36].

Study revealed that limited awareness and widespread misinformation about vaccination constitute significant barriers to childhood immunization uptake [37]. Some participants demonstrated inadequate awareness regarding the purpose, schedule, and benefits of vaccines, whereas others held misconceptions about potential side effects and safety. These misunderstandings often led to fear, hesitation, and, in some cases, refusal to vaccinate their children, ultimately contributing to ZD and underimmunization. The study explored prevailing traditional beliefs and social norms that assign sole responsibility for child vaccination to mothers. This finding is in line with studies conducted in Ethiopia [11, 31], a systematic review finding in Sub‐Saharan Africa [35], and Latin America [38].

### 4.1 Implications for Policy

The findings of the current study are directly relevant to achieving SDG targets 3.2, 3.3, and 3.8 aimed at ending preventable deaths of newborns and children under 5 years of age; ending epidemics of communicable diseases; and achieving universal health coverage, including access to safe, effective vaccines, by 2030, respectively [39]. Policymakers should prioritize context‐specific, culturally sensitive health education campaigns that address misinformation and normalize mild post‐vaccination reactions. The identified geographic and access barriers suggest the importance of policies that improve equitable access to immunization services by expanding outreach services, strengthening mobile vaccination programs, and improving the distribution of health facilities in underserved rural areas. The role of family and social support, particularly male partner involvement, underscores the need for policies promoting male engagement in maternal and child health programs.

### 4.2 Implications for Practice

HEWs should provide the targeted and continuous health education focusing on benefits of immunization, vaccination schedules, and management of common side effects. Moreover, efforts should also be made to actively dispel myths and misconceptions among mothers, caregivers, and parents, including the male partners, through community dialogues, testimonials, and peer education.

### 4.3 Strengths and Limitations

This study had strengths. The use of multiple qualitative methods (FGDs, IDIs, and KIIs) allowed for data triangulation, enhancing the credibility and depth of the findings [40]. The inclusion of different participant groups, including mothers, mothers/caregivers of ZD children, and health decision‐makers at different levels, provided diverse perspectives on immunization practices. Despite its strengths, this study had some limitations. First, as a qualitative study, the findings are not generalizable to all populations, although they provide valuable in‐depth understanding. Second, the study may be subject to social desirability bias, as participants might have provided responses they perceived as acceptable, particularly regarding health practices. Third, no theoretical framework was used to guide this study.

## 5 Conclusion

This study highlights that childhood immunization uptake in central Ethiopia is shaped by a complex interaction of individual, social, and health‐system factors. Positive maternal attitudes and beliefs toward immunization support from family members, and caregivers’ sense of responsibility emerged as key facilitators of immunization. In contrast, fear and misconceptions about vaccines, inadequate awareness and misinformation, health‐system and access constraints, and being overwhelmed with household chores and responsibilities were considered major barriers to immunization. Interventions that strengthen community awareness, counter misinformation, enhance family and social support, and improve the accessibility and responsiveness of immunization services are helpful to improve the immunization uptake. This implies that the childhood immunization uptake requires integrated and multi‐level interventions in the central Ethiopian region.

## Author Contributions

Zeleke Dutamo Agde, Amanuel Nuramo, and Belayneh Hamdela Jena conceptualized the idea and the design and wrote up the draft manuscript. Zeleke Dutamo Agde, Temesgen Abdi, and Tesfaye Lejisso were involved in data analysis. Samuel Darge, Ashenafi Petros, Temesgen Kelaye, Legese Petros, Belayneh Hamdela Jena, Amanuel Nuramo, and Mamush Hussein were involved in critically reviewing and revising the manuscript. All authors read and approved the final version of the manuscript.

## Funding

The authors have nothing to report.

## Ethics Statement

Ethical approval was obtained from the Institutional Research Ethics Committee (IREC) of the Central Ethiopia Region Public Health Institute on October 2, 2025 (CEPHI‐IRC‐004‐2025), in accordance with the World Medical Association Declaration of Helsinki [41]. A permission letter was obtained from the zonal health department, district health offices, and kebele administration prior to data collection. The data collectors explained the purpose of the study and its procedures. They also explained the participants’ right not to participate or withdraw from the study at any time as explained in the information sheet. Confidentiality of the information was ensured through anonymous coding of the data, and all the information they provide is not shared with anyone. The consolidated criteria for reporting qualitative research (COREQ): A 32‐item checklist was used to report the study findings [42].

## Consent

Written informed consent was obtained from all study participants.

## Conflicts of Interest

The authors declare no conflicts of interest.

## Supporting information



Supporting File 1: puh270456‐sup‐0001‐SuppMat.doc

Supporting File 2: puh270456‐sup‐0002‐SuppMat.docx

## Data Availability

The datasets used and/or analyzed during the current study are available from the corresponding author on reasonable request.
