# Supplementary material for: Barriers to and Facilitators of Childhood Immunization in Central Ethiopia Region: A Qualitative Study
Source: Public Health Chall. 2026 Nov 7;5(4):e70456. doi: 10.1002/puh2.70456 (PMC13744627; doi:10.1002/puh2.70456)
Supplement: Supplementary file 1 [file PUH2-5-e70456-s001.docx]

# Semi structured topic guide

1. Can you tell me a little about yourself (age, role in the household/community, number of children)?
2. What is your experience with child immunization in your household/community?
3. What do you know about childhood immunization?
4. How do people in this community generally feel about immunization?
5. Do you believe vaccines are effective and safe? Why or why not?
6. Are there any common misconceptions or rumors about vaccines in your area?
7. What are the main challenges mothers/caregivers faces in getting their children vaccinated?
   - Probe: Distance to health facility, transport cost, clinic hours, stock-outs, workload at home, etc.
9. Are there cultural, religious, or traditional beliefs that discourage immunization in your community?
10. How do health workers treat mothers/caregivers who bring their children for immunization?
11. Are there any side effects of vaccines that worry mothers/caregivers?
13. What encourages or motivates mothers/caregivers to vaccinate their children?
14. How helpful is the information provided by health extension workers or health professionals?
15. Have you received reminders (e.g., from HEWs, mobile messages, announcements) about vaccination schedules?
16. What roles do fathers/partners and other family members play in decision-making about child immunization?
18. What do you think can be done to improve child immunization coverage in this area?
19. How can the health system or community better support caregivers to complete vaccination schedules?
20. What kind of information, support, or services would help increase acceptance and accessibility of vaccines?
21. Is there anything else you would like to add about immunization in your community?

**Thank the participant(s) for their time and contributions.**
